# Obesity Metabolic Phenotype, Changes in Time and Risk of Diabetes Mellitus in an Observational Prospective Study on General Population

**DOI:** 10.3389/ijph.2022.1604986

**Published:** 2022-09-29

**Authors:** Chan Yang, Xiaowei Liu, Yuanyuan Dang, Juan Li, Jingyun Jing, Di Tian, Jiangwei Qiu, Jiaxing Zhang, Ni Yan, Xiuying Liu, Yi Zhao, Yuhong Zhang

**Affiliations:** ^1^ Department of Epidemiology and Health Statistics, School of Public Health and Management, Ningxia Medical University, Yinchuan, China; ^2^ Department of Community Nursing, School of Nursing, Ningxia Medical University, Yinchuan, China; ^3^ Department of Nutrition and Food Hygiene, School of Public Health and Management, Ningxia Medical University, Yinchuan, China; ^4^ Key Laboratory of Environmental Factors and Chronic Disease Control, Ningxia Medical University, Yinchuan, China

**Keywords:** obesity, type 2 diabetes, metabolic abnormalities, metabolically healthy overweight/obese, metabolically unhealthy overweight/obese

## Abstract

**Objectives:** To evaluate the distribution and changes in different obesity metabolic phenotypes, as well as their impact on the incidence of type 2 diabetes mellitus (T2DM) in a northwest Chinese population sample.

**Methods:** Data comes from prospective cohort study (*n* = 1,393, mean follow up = 9.46 years). Participants were classified into four groups through a combination of the Chinese Diabetes Society (CDS) diagnostic criteria for metabolic syndrome with anthropometric measurements: metabolically healthy normal weight (MHNW), metabolically healthy overweight/obese (MHO), metabolically unhealthy normal weight (MUNW), and metabolically unhealthy overweight/obese (MUO). Cox regression models with time-dependent covariates were used to evaluate changes in obesity metabolic phenotypes and risk of T2DM.

**Results:** Participants in MUO state had the highest risk of developing T2DM, the incidence density was 12.10/1,000 person-year. The MHO and MUO groups showed an increased risk of incident diabetes based on body mass index (BMI) (HR, 1.29; 95% CI, 1.03–1.61; *p* = 0.026 and HR, 1.20; 95% CI, 1.02–1.40; *p* = 0.024 respectively.) Besides, the MHO group had an increased risk of incident diabetes based on waist circumference (WC) (HR, 1.41; 95% CI, 1.10–1.80; *p* = 0.006).

**Conclusion:** Diabetes is more frequent in the MHO and MUO groups and co-occurrence of obesity and metabolic abnormalities (MA) contributes to the development of T2DM.

## Introduction

Obesity and overweight are major clinical and public health issues worldwide. In 2020, the World Health Organization (WHO) reported that according to body mass index (BMI) 1.9 billion adults (>18 years) were overweight, with over 650 million were obese [1]. Over the past decade, the number of overweight and obese people has reached pandemic levels and the prevalence of obesity has expanded worldwide [2]. Obesity has been frequently linked to occurrence of metabolic disorders like diabetes mellitus, dyslipidemia, hypertension, insulin resistance, and cardiovascular disease [3–5].

The prevalence of diabetes has increased worldwide. According to the International Diabetes Federation (IDF), there were 351.7 million people of working age (20–64 years) with diabetes in 2019, this number is expected to reach 417.3 million by 2030 [6]. Obesity is a significant risk factor for the development of insulin resistance and type 2 diabetes. Non-esterified fatty acids, glycerol, hormones, pro-inflammatory cytokines, and other factors released by adipose tissue contribute to insulin resistance in obese people [7]. Obesity, characterized by the accumulation of excessive body fat, is associated with a variety of metabolic abnormalities (MA). MA generally include hypertension, hyperglycemia and dyslipidemia. However, not all obese people have Metabolic abnormalities, and not all people with MA are obese [8, 9].

Obesity and MA are common coexisting conditions which contribute to other diseases. Research has attempted to define obesity metabolic phenotypes according to metabolic abnormalities co-occurrence. Previous research indicates that some overweight/obese individuals have a healthy metabolic status, and this subtype is linked to a lower risk of obesity-related diseases [10, 11]. As such, researchers have focused on investigating metabolically healthy overweight/obese subtype [12–19]. Literature also shows that diabetes is differently associated to different phenotypes. In the San Antonio Heart Study (*n* = 3,700 participants free of diabetes at baseline) both metabolically unhealthy normal weight (MHNW) and metabolically healthy obese (MHO) individuals had an increased diabetes risk (median follow up = 7.4 years) [12]. In the Binhai Health Study (*n* = 49,702 community dwelling elderly participants without diabetes at baseline) MHO was associated with an increased incidence of diabetes (follow up = 4 years) [13]. In the CoLaus study (n = 3,038 participants free from metabolic syndrome and cardiovascular disease at baseline) MHO participants were significantly more likely to develop type 2 diabetes mellitus (T2DM) than normal-weight people with abnormal metabolic profiles (follow up = 10.9 years) [14]. Other studies have obtained similar outcomes [15, 16]. However, the results differ across different countries and regions [17–19]. For example, in the community-based Uppsala Longitudinal Study of Adult Men study (*n* = 1,675 participants without diabetes at baseline) overweight or obese men without metabolic syndrome were at increased risk for diabetes (follow up = 20 years) [17]. These inconsistencies could be attributed to differences in the classification criteria for obesity metabolic phenotypes, as well as differences in lifestyle, dietary habits, obesity distribution, and follow-up periods across regions.

This study, therefore, aimed to describe the distribution and changes in different obesity metabolic phenotypes, as well as examine their effects on the incidence of T2DM after a 9.46-year follow-up in an adult northwest Chinese population-based sample.

## Methods

### Subjects and Data Collection

**FIGURE 1:**
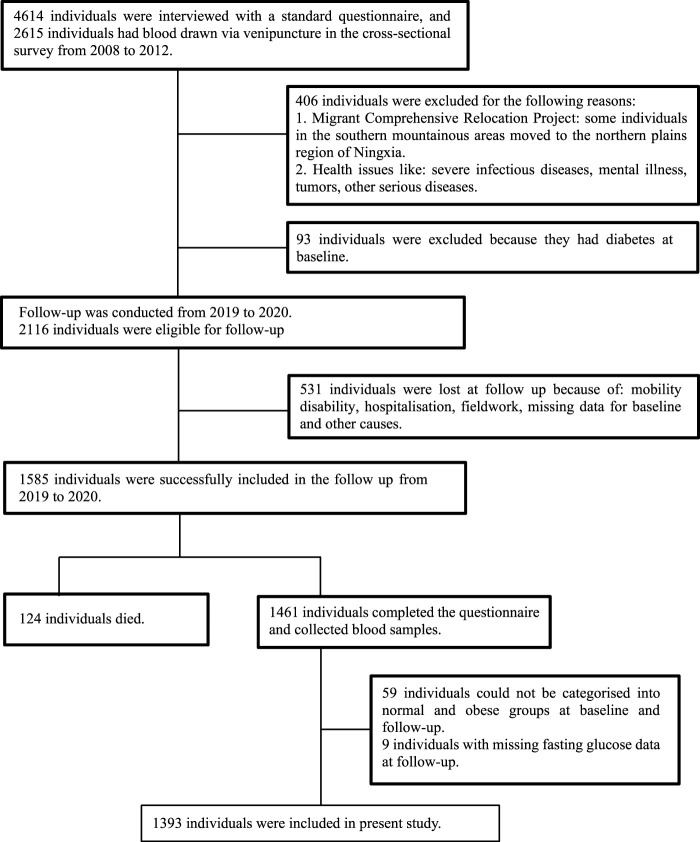
Flow chart for participants recruited (Obesity Metabolic Phenotype, Changes in Time and Risk of Diabetes Mellitus in an Observational Prospective Study on General Population, Ningxia, China. 2008-2020).

Data from the first stage of China North-west Cohort (CNC-NX) were analysed in this study. Data were collected from a cross-sectional survey conducted among rural residents in the Ningxia region of northwest China between 2008 and 2012, all subjects in this study were adults aged 18–76 years. In the cross-sectional survey, 4614 individuals were interviewed using a standard questionnaire, and 2615 individuals had blood drawn *via* venipuncture [20]. Then our research group completed a longitudinal follow-up survey of the initial subjects between 2019 and 2020. In total 1,585 individuals were successfully followed up, of these 124 had died and of the remaining 1,461, 1,393 subjects were included. Specific schematic process of inclusion and exclusion criteria can be found in Figure 1.

This study was conducted in accordance with the guidelines of the Declaration of Helsinki and approved by the ethics review board of Ningxia Medical University. All participants provided informed consent and signed a consent letter following an explanation of the study objectives and methods.

### Data Collection and Measurements

All participants completed a questionnaire survey, which was administered through in-person interviews. The investigators were trained before the surveys, and both the baseline and follow up questionnaire had similar content. Questionnaires included demographic information (age, gender, educational attainment, and marital status), lifestyle habits (smoking, tea, and alcohol consumption status, physical exercise), and medical history. Smoking was defined as ≥1 cigarette/day sustained for ≥6 months. Alcohol consumption was defined as ≥1 time per week for ≥6 months. Drinking tea was defined as consuming at least one cup per week for more than 6 months. Physical exercise was defined as exercising at least three times per week for more than 30 min each time. Anthropometric measurements included height, weight, waist circumference (WC), and hip circumference (HC). Height, WC, and HC at baseline were measured using a portable ruler. Bodyweight was measured using a weight scale (Omron, China). An automated Blood pressure (BP) monitor (OMRON HEM model) was used to measure brachial BP after a 5-min rest. The same methods were used for the measurement of height, weight, WC, HC and BP at follow-up. Body Mass Index (BMI) was calculated as weight (kg)/height (m)^2^. An automated blood pressure monitor was used to measure brachial blood pressure after a 5-min rest period.

Blood samples were taken in the morning after an 8-hour fast. At baseline, fasting blood glucose (FBG) levels were measured using a One Touch Ultra 2 (Life Scan, USA); serum insulin levels were measured using the enzyme-linked immune chemiluminescence method; total cholesterol (TC), triglyceride (TG), and high-density lipoprotein cholesterol (HDL-C) were determined by the enzymatic method (CHOD-PAP, Roche Diagnostics GmbH); low-density lipoprotein cholesterol (LDL-C) was calculated using the Friedewald formula [21]. During the follow-up survey, TC, TG, HDL-C, LDL-C, and FBG levels were measured using biochemical auto-analyzers (Mindray BS-430, Shenzhen, China); serum insulin (FINS) concentrations were measured with a chemiluminescence immunoassay analyser (Mindray CL-2000i, Shenzhen, China).

The homeostatic model assessment (HOMA) index was calculated from fasting glucose and insulin concentrations to assess insulin resistance (IR) and β-cell function [22]. These indices were calculated using the following formula: HOMA-IR = (fasting glucose [mmol/L] × fasting insulin [µIU/mL])/22.5; HOMA-β = 20 × fasting insulin (µIU/ml)/(fasting glucose [mmol/L] – 3.5).

### Definitions

MA was defined using the most recent China Diabetes Society (CDS) criteria for metabolic syndrome [23]. Overweight and obesity were defined according to BMI and WC criteria published by the adult weight determination criteria brought out by the National Health Commission of the People’s Republic of China [24]. We performed a pairwise combination between MA and obesity status. Participants were classified as metabolically healthy normal weight (MHNW), metabolically healthy overweight/obese (MHO), metabolically unhealthy normal weight (MUNW), and metabolically unhealthy overweight/obese (MUO). Details are displayed in Table 1. T2DM was diagnosed using the Guidelines for the Prevention and Control of Type 2 Diabetes in China (2017 Edition) [23].

**TABLE 1:** The judgment criteria of metabolic abnormalities, overweight and obesity (Obesity Metabolic Phenotype, Changes in Time and Risk of Diabetes Mellitus in an Observational Prospective Study on General Population, Ningxia, China. 2008-2020).

MA (at least one of the following requirements)	Overweight and obesity	
	Defined as BMI	Defined as WC
➢ Hyperglycemia FBG ≥6.1 mmol/L, or 2-h plasma glucose levels ≥7.8 mmol/L after a 75-g oral glucose tolerance test (OGTT), or have been diagnosed with diabetes	➢ Normal weight: 18.5 ≤ BMI <24 kg/m^2^	➢ Normal weight: WC <90 cm for men and <85 cm for women
➢ Hypertension have BP ≥130/85 mmHg, or have been diagnosed with hypertension	➢ Overweight/obesity: BMI ≥24 kg/m^2^	➢ Overweight/obesity (Central obesity): WC ≥ 90 cm for men and ≥85 cm for women
➢ Dyslipidemia have TG ≥1.7 mmol/L, or HDL-C < 1.04 mmol/L		

MA, metabolic abnormalities; BMI, body mass index; WC, waist circumstance; BP, blood pressure; TC, total cholesterol; TG, triglyceride; HDL-C, high-density lipoprotein cholesterol; LDL-C, low-density lipoprotein cholesterol; FBG, fasting blood-glucose.

### Statistical Analysis

A descriptive statistical analysis was conducted. Continuous variables were presented as mean ± standard ( $\bar{X}$ ±S), whereas categorical variables were presented as frequencies and percentages (N [%]). The chi-square test or one-way analysis of variance (ANOVA) was used to make comparisons between categorical and continuous variables. Missing data were imputed by multiple imputations. The study endpoint was the occurrence of diabetes (fasting glucose ≥7.0 mmol/L). Person years were calculated from the baseline survey date to the follow-up date to estimate incidence rates. Diabetes incidence was calculated per 1,000 years, based on the number of people who developed diabetes during the follow-up as the numerator and total person-time as the denominator. Hazard ratios (HRs) and 95% confidence intervals (CIs) were calculated using time-dependent covariates of Cox regression models. Cox regression models were used to examine the effects of covariates on time-to-events. Three multivariate models were developed: Model 1 was unadjusted; Model 2 was adjusted for sex and age; Model 3 was adjusted for education level, marital status, smoking status, alcohol drinking status, tea-drinking status, and physical exercise; Model 4 was adjusted for BMI/WC, HC, SBP, DBP, TC, TG, HDL-C, LDL-C, and HOMA-β. Statistical analysis was performed with STATA 16.0 (StataCorp LLC, Texas, USA).

## Results

Table 2 shows the baseline characteristics of the study participants in terms of metabolic health and obesity status. The study enlisted the participation of 1,393 individuals. There were 574 men (mean age, 50 years; age range, 19–76 years) and 819 women (mean age, 47 years; age range, 22–76 years). Overweight/obesity was defined at each BMI>24 kg/m^2^ and WC at each >90 for men and 85 for women. Obesity metabolic phenotype based on BMI classification created the following groups: 316 (22.68%) in the MHNW group, 114 (8.18%) in the MHO group, 500 (35.89%) in the MUNW group, and 463 (33.24%) in the MUO group. Obesity metabolic phenotypes based on WC classification created the following groups: 338 (24.26%) were in the MHNW group, 92 (6.60%) in the MHO group, 600 (43.07%) in the MUNW group, and 363 (26.06%) in the MUO group. The same situation happened in both classifications, the four groups had statistically significant differences. Among all groups, those in the MHNW group were the youngest. MHO participants were less likely to be smokers, and had intermediate levels of diabetes risk when compared to the MUNW and MUO groups. There were statistically significant differences in age, sex, education level, WC/BMI, HC, BP, and serum lipid and blood glucose levels between the four groups. There were no significant differences in marital status, smoking status, alcohol, and tea-drinking status, physical activity, and serum FINS among the four groups.

**TABLE 2:** Baseline characteristics of participants in different metabolic obesity phenotypes (Obesity Metabolic Phenotype, Changes in Time and Risk of Diabetes Mellitus in an Observational Prospective Study on General Population, Ningxia, China. 2008-2020).

Characteristics	MHNW	MHO	MUNW	MUO	*p*-value
Definitions based on BMI					
N (%)	316 (22.68)	114 (8.18)	500 (35.89)	463 (33.24)	
Sex					0.046
Men	127 (40.19)	44 (38.60)	230 (46.00)	173 (37.37)	
Women	189 (59.81)	70 (61.40)	270 (54.00)	290 (62.63)	
Education levels					0.001
Illiteracy	114 (36.08)	39 (34.21)	203 (40.60)	229 (49.46)	
Elementary school	102 (32.28)	47 (41.23)	147 (29.40)	117 (25.27)	
Middle school and above	100 (31.64)	28 (24.56)	150 (30.00)	117 (25.27)	
Marital status					0.549
Unmarried	11 (3.48)	3 (2.63)	25 (5.00)	16 (3.46)	
Married	305 (96.52)	111 (97.37)	475 (95.00)	447 (96.54)	
Smoking status					0.262
Yes	59 (18.67)	12 (10.53)	87 (17.40)	66 (14.25)	
No	257 (81.33)	99 (86.84)	413 (82.60)	397 (85.75)	
Alcohol drinking status					0.953
Yes	28 (8.86)	10 (8.77)	45 (9.00)	46 (9.94)	
No	288 (91.14)	104 (91.23)	455 (91.00)	417 (90.06)	
Tea drinking status					0.273
Yes	174 (55.06)	64 (56.14)	257 (51.40)	226 (48.81)	
No	142 (44.94)	50 (43.86)	243 (48.60)	237 (51.19)	
Physical exercise					0.931
Yes	18 (5.70)	7 (6.14)	29 (5.80)	31 (6.70)	
No	298 (94.30)	107 (93.86)	471 (94.20)	432 (93.30)	
Mean ± SD					
Age (years)	45.66 ± 0.68	48.73 ± 1.11	49.02 ± 0.50	49.79 ± 0.48	<0.001
WC (cm)	76.48 ± 2.18	85.15 ± 0.73	77.82 ± 1.23	87.54 ± 0.33	<0.001
HC (cm)	88.84 ± 0.24	96.14 ± 0.47	89.86 ± 0.18	96.85 ± 0.23	<0.001
SBP (mmHg)	111.61 ± 0.77	117.54 ± 1.69	127.37 ± 0.86	133.05 ± 0.90	<0.001
DBP (mmHg)	70.35 ± 0.51	72.97 ± 1.11	81.47 ± 0.48	84.75 ± 0.51	<0.001
FBG (mmol/L)	4.57 ± 0.14	4.19 ± 0.30	5.51 ± 0.07	5.57 ± 0.08	<0.001
FINS (µIU/ml))	5.84 ± 0.23	5.79 ± 0.30	6.32 ± 0.34	6.34 ± 0.19	0.542
TC (mmol/L)	3.67 ± 0.07	3.75 ± 0.09	3.87 ± 0.03	4.06 ± 0.05	<0.001
TG (mmol/L)	0.81 ± 0.05	0.81 ± 0.08	1.27 ± 0.03	1.58 ± 0.05	<0.001
HDL-C (mmol/L)	1.42 ± 0.03	1.39 ± 0.03	1.29 ± 0.02	1.23 ± 0.02	<0.001
LDL-C (mmol/L)	1.87 ± 0.05	1.98 ± 0.07	2.01 ± 0.03	2.11 ± 0.04	<0.001
HOMA–IR	1.20 ± 0.07	1.10 ± 0.10	1.55 ± 0.09	1.58 ± 0.05	0.001
HOMA–β	82.62 ± 4.01	89.47 ± 7.71	65.81 ± 3.69	63.22 ± 2.63	<0.001
Definitions based on WC					
N (%)	338 (24.26)	92 (6.60)	600 (43.07)	363 (26.06)	
Sex					<0.001
Men	121 (35.80)	50 (54.35)	226 (37.67)	177 (48.76)	
Women	217 (64.20)	42 (45.65)	374 (62.33)	186 (51.24)	
Education levels					0.003
Illiteracy	116 (34.32)	37 (40.22)	256 (42.67)	176 (48.48)	
Elementary school	115 (34.02)	34 (36.96)	163 (27.17)	101 (27.82)	
Middle school and above	107 (31.66)	21 (22.83)	181 (30.17)	86 (23.69)	
Marital Status					0.520
Unmarried	12 (3.55)	2 (2.17)	22 (3.67)	19 (5.23)	
Married	326 (96.45)	90 (97.83)	578 (96.33)	344 (94.77)	
Smoking status					0.525
Yes	58 (17.16)	16 (17.39)	88 (14.67)	65 (17.91)	
No	280 (82.84)	76 (82.61)	512 (85.33)	298 (82.09)	
Alcohol drinking status					0.123
Yes	26 (7.69)	12 (13.04)	49 (8.17)	42 (11.57)	
No	312 (92.31)	80 (86.96)	551 (91.83)	321 (88.43)	
Tea drinking status					0.222
Yes	186 (55.03)	52 (56.52)	293 (48.83)	190 (52.34)	
No	152 (44.97)	40 (43.48)	307 (51.17)	173 (47.66)	
Physical exercise					0.232
Yes	16 (4.73)	9 (9.78)	34 (5.67)	26 (7.16)	
No	322 (95.27)	83 (90.22)	566 (94.33)	337 (92.84)	
Mean ± SD					
Age (years)	44.83 ± 0.64	52.53 ± 1.09	47.67 ± 0.44	52.24 ± 0.51	<0.001
BMI	21.91 ± 0.11	25.50 ± 0.32	22.77 ± 0.09	26.35 ± 0.14	<0.001
HC (cm)	89.35 ± 0.25	96.08 ± 0.56	90.54 ± 0.17	97.63 ± 0.27	<0.001
SBP (mmHg)	111.91 ± 0.79	117.89 ± 1.88	126.89 ± 0.78	135.39 ± 1.01	<0.001
DBP (mmHg)	70.54 ± 0.53	72.92 ± 1.16	81.63 ± 0.44	85.39 ± 0.58	<0.001
FBG (mmol/L)	4.62 ± 0.15	3.94 ± 0.31	5.50 ± 0.07	5.60 ± 0.08	<0.001
FINS (µIU/ml))	5.90 ± 0.22	5.58 ± 0.35	6.02 ± 0.14	6.85 ± 0.48	0.708
TC (mmol/L)	3.66 ± 0.05	3.78 ± 0.11	3.85 ± 0.03	4.15 ± 0.05	<0.001
TG (mmol/L)	0.81 ± 0.04	0.81 ± 0.09	1.26 ± 0.03	1.67 ± 0.06	<0.001
HDL-C (mmol/L)	1.42 ± 0.02	1.39 ± 0.04	1.27 ± 0.02	1.24 ± 0.02	<0.001
LDL-C (mmol/L)	1.87 ± 0.04	2.02 ± 0.08	2.01 ± 0.03	2.14 ± 0.04	<0.001
HOMA–IR	1.22 ± 0.07	0.99 ± 0.11	1.48 ± 0.04	1.71 ± 0.12	<0.001
HOMA–β	82.16 ± 3.98	92.91 ± 8.03	62.98 ± 2.27	67.20 ± 4.89	<0.001

Values are presented as the mean ± SD or number (%). *p*-value obtained in the ANVOA or chi-squared test. MHNW, metabolically healthy normal weight; MHO, metabolically healthy overweight/obese; MUNW, metabolically unhealthy normal weight; MUO, metabolically unhealthy overweight/obese; BMI, body mass index; WC, waist circumstance; HC, hip circumstance; SBP, systolic blood pressure; DBP, diastolic blood pressure; TC, total cholesterol; TG, triglyceride; HDL-C, high-density lipoprotein cholesterol; LDL-C, low-density lipoprotein cholesterol; FBG, fasting blood-glucose; FINS, fasting insulin; HOMA-IR, homeostasis model assessment of insulin resistance; HOMA-β, homeostasis model assessment of β cell function.

According to the BMI definition, the baseline proportions of MHNW, MHO, and MUNW were higher than in the follow-up proportions, while the proportions of MUO phenotypes were lower. Similarly, for WC-based definitions, the baseline proportions of MHNW, MHO, and MUNW were higher compared to the follow-up, while the proportions of MUO phenotypes were lower (Supplementary Table S1).

At baseline, the proportions of obese individuals based on BMI and WC were 41.20 and 32.66%, respectively, and at follow-up, the proportions were 64.47 and 59.94%, respectively. The proportions of the MHNW, MHO, and MUNW phenotypes were higher at both baseline and follow-up, while the proportions of the MUO phenotypes were lower when using the BMI definitions compared with WC definitions (Supplementary Figure S1).

The average follow-up lasted for 9.46 years (range, 6.75–12.17 years). Metabolic obesity phenotypes changed over time, and we looked at what proportion of baseline phenotypes changed to other phenotypes during the follow-up period (Table 3). For BMI-based definitions, 666 (47.81%) of participants showed no changes in phenotypes compared with baseline. Of the subjects that had been classified as MHNW at follow-up, 10.76% belonged to the MHO category at baseline, 42.09% to MUNW and 33.23% to MUO. In the MUO group at baseline, only 1.51% of the individuals eventually changed to MHNW. Obesity metabolic phenotypes based on WC classification, 576 (58.65%) participants showed no changes in phenotypes when compared to the baseline. In the MHNW group, 8.28% of individuals were MHO, 40.83% were MUNW, and 34.62% were MUO. In the MUO group, only 1.38% of the individuals were MHNW.

**TABLE 3:** Changes in obesity metabolic phenotype from baseline to follow-up (Obesity Metabolic Phenotype, Changes in Time and Risk of Diabetes Mellitus in an Observational Prospective Study on General Population, Ningxia, China. 2008-2020).

Baseline		Follow-up			
		MHNW	MHO	MUNW	MUO
Definitions based on BMI		101 (100)	90 (100)	394 (100)	808 (100)
MHNW	316 (100)	44 (13.92)	34 (10.76)	133 (42.09)	105 (33.23)
MHO	114 (100)	2 (1.75)	10 (8.77)	6 (5.26)	96 (84.22)
MUNW	500 (100)	48 (9.60)	21 (4.20)	218 (43.60)	213 (42.60)
MUO	463 (100)	7 (1.51)	25 (5.40)	37 (7.99)	394 (85.10)
Definitions based on WC		116 (100)	75 (100)	442 (100)	760 (100)
MHNW	338 (100)	55 (16.27)	28 (8.28)	138 (40.83)	117 (34.62)
MHO	92 (100)	3 (3.26)	4 (4.35)	10 (10.87)	75 (81.52)
MUNW	600 (100)	53 (8.83)	28 (4.67)	234 (39.00)	285 (47.50)
MUO	363 (100)	5 (1.38)	15 (4.13)	60 (16.53)	283 (77.96)

MHNW, metabolically healthy normal weight; MHO, metabolically healthy overweight/obese; MUNW, metabolically unhealthy normal weight; MUO, metabolically unhealthy overweight/obese; BMI, body mass index; WC, waist circumstance.

**FIGURE 2:**
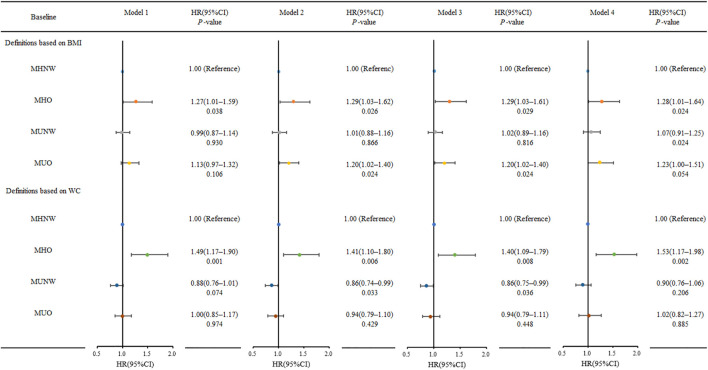
Risk for incidence diabetes according to metabolic obesity phenotypes at 9.46-year follow-up (Obesity Metabolic Phenotype, Changes in Time and Risk of Diabetes Mellitus in an Observational Prospective Study on General Population, Ningxia, China. 2008-2020). Model 1 without adjusted; Model 2 adjusted for sex, age; Model 3 adjusted for Model 2 plus education level, marital status, smoking status, alcohol drinking status, tea dinking status, physical exercise; Model 4 adjusted for Model 3 plus BMI/WC, HC, SBP, DBP, TC, TG, HDL-C, LDL-C, and HOMA-β.

During the follow-up period, 117 (8.40%) participants developed type 2 diabetes. The MHNW phenotype served as the control group. We looked at the risk of incident diabetes based on BMI, WC, and metabolic status. The incidence of diabetes and adjusted HRs are shown in Table 4 and Figure 2. The MUO group had the highest risk of incident diabetes compared to the reference group MHNW, followed by the MHO and MUNW groups. The definitions based on BMI and the incidence of diabetes per 1,000 person-years for MHNW, MHO, MUNW, and MUO group participants were 4.96, 9.92, 7.30, and 12.10, respectively. Diabetes was most prevalent in the MUO group at baseline. The MHNW phenotype was used as the control group. Without adjustment, the MHO group (HR, 1.27; 95% CI, 1.01–1.59, *p* = 0.038) had a higher risk of incident diabetes. The MHO group (HR, 1.29; 95% CI, 1.03–1.61, *p* = 0.026) and MUO group (HR, 1.20; 95% CI, 1.02–1.40, *p* = 0.024) had an increased risk of incident diabetes after adjusting for sex and age. After adjusting for demographics, lifestyle, anthropometric parameters, and laboratory test results, a reanalysis of the HRs in the MHO and MUO groups revealed similar trends (Figure 2). Obesity metabolic phenotypes based on WC classification, diabetes incidence per 1,000 person-years for MHNW, MHO, MUNW, and MUO participants was 5.77, 8.17, 6.74, and 14.81, respectively. Without adjustment, the MHO group (HR, 1.49; 95% CI, 1.17–1.90, *p* = 0.001) had a higher risk of incident diabetes. With further adjustment, the *p*-values were less than 0.05. Figure 2 depicts the results of the other three models in the MHO group, which showed similar trends. Weight gain in adults with a metabolically healthy phenotype increased the risk of diabetes, unhealthy metabolic status, and weight gain, and increased the risk of diabetes in adults.

**TABLE 4:** Incidence rate of diabetes by metabolic obesity phenotypes at baseline (Obesity Metabolic Phenotype, Changes in Time and Risk of Diabetes Mellitus in an Observational Prospective Study on General Population, Ningxia, China. 2008-2020).

	MHNW	MHO	MUNW	MUO
Definitions based on BMI				
Cases	316	114	500	463
Incident cases	15	10	37	55
Person-years	3,021.88	1,007.98	5,071.46	4545.29
Incidence density (per 1,000 person years)	4.96	9.92	7.30	12.10
Definitions based on WC				
Cases	338	92	600	363
Incident cases	19	6	42	50
Person-years	3,295.04	734.82	6,239.68	3,377.07
Incidence density (per 1,000 person years)	5.77	8.17	6.74	14.81

MHNW, metabolically healthy normal weight; MHO, metabolically healthy overweight/obese; MUNW, metabolically unhealthy normal weight; MUO, metabolically unhealthy overweight/obese; BMI, body mass index; WC, waist circumstance.

## Discussion

Our research is based on a population-based prospective observational study with an average follow-up period of 9.46 years. To define MA, we chose standards appropriate for Chinese people. Taking into account the distinction between central and systemic obesity, the authors described the distribution and changes in different obesity metabolic phenotypes and examined the effects of different phenotypes on the incidence of T2DM.

A total of 1,393 subjects were grouped and analysed according to their baseline metabolic obesity status. Notably, 117 (8.4%) of those who were overweight/obese and had no diabetes at baseline developed T2DM. More than half of the subjects (500 in the MUNW group, and 463 in the MUO group) had MUNW and MUO phenotypes at baseline, with MUNW accounting for the same percentage as MUO. Furthermore, MHO and MUO phenotypes were linked to an increased risk of developing T2DM in individuals. Of note, only half of the study subjects retained the same phenotype and were mostly concentrated in the MUO during follow-up. Time-dependent Cox regression analysis demonstrated an association of obesity with an increased risk of diabetes. Contrarily, in the present study, MHO was not regarded as a healthy development in rural middle-aged and elderly populations.

At baseline, the MA participants had higher percentages of all participants in this study, up to 69%, while the MHO group had a small number of individuals. The number of metabolically healthy participants decreased during the follow-up period, particularly in the MHNW group. As a result, the number of MUO phenotypes increased. Compared with other studies [17, 25], the proportion of people in our study who had metabolic health was relatively low, particularly in the MHO phenotype. MH was defined differently in different studies based on different criteria, whereas MA included one [26, 27] or more [12, 16, 17, 25] of the following abnormalities, lipid metabolism, glucose metabolism, or blood pressure, which may have influenced the results. Another cohort study of 11,865 Chinese adults defined metabolically healthy as participants who met <2 of normal BP, TG, HDL-C, FPG (follow up = 6 years) [27]. In the San Antonio Heart Study, Aung et al. defined MHNW as individuals with normal weight and two or more metabolic abnormalities [12]. A prospective cohort study of 381,363 UK Biobank participants also designated participants who fulfilled at least four metabolically healthy criteria were considered metabolically healthy, and these elements included BP, C-reactive protein, triacylglycerols, LDL-C, HDL-C and HbA1c (follow up = 11.2 years) [16]. Furthermore, participants in our study were older than in previous studies [12, 15, 26]. Given the long follow up of our study, most of the participants progressed from middle age to old age, and this could have promoted a change in work activities. In fact, the recruited population was rural, thus their activities were mostly agricultural, and with increasing age, it is expected that also agricultural activities may have shifted to lighter activities like light agricultural labour or domestic household work. This potentially decreases total calorie consumption and weight gain. Our follow-up coincided with the perimenopause of the majority of female participants, and oestrogen levels of women changed with menopause. Postmenopausal women are more likely to be in an inflammatory state, which promotes the progression of chronic inflammatory diseases [28]. Obesity is a chronic low-grade inflammatory state that has been linked to a critical predisposing factor for MA development [29]. Obesity, inflammation, and abnormal metabolic progression are all caused by changes in oestrogen levels.

As such, there is a critical need to encourage middle-aged and elderly people to enhance the understanding of health and chronic diseases in a variety of ways, place much focus on the reasons why people become more obese and more metabolically unhealthy, and consult both general practitioners and endocrinologists on a case-by-case basis. Above that, avoiding or even reversing phenotype transformation will be the crucial point of chronic disease prevention.

Half of the participants had a stable metabolic obesity phenotype, mainly the MUO phenotype. Most people in the MUO phenotype population remained in the same unhealthy state at follow-up. Only a small proportion of MHNW patients remained healthy at the end of the follow-up period. The MUNW phenotype was also unstable, with approximately 40% of participants switching to MUO. In other words, only a few people remained healthy, while the majority of people developed obesity and MA. MHO was the most easily altered phenotype. The disparities between MHNW and MHO most likely reflected the negative effects of obesity. Compelling evidence shows that MHO participants are in an intermediate and temporary state in the transformation of metabolically healthy to unhealthy and metabolic-related diseases [27, 30]. Therefore, it is critical for all individuals to maintain a healthy weight to avoid phenotypic changes.

Diabetes risk may differ between obesity metabolic phenotypes [31]. Based on BMI criteria, we discovered that the MHO and the MUO groups had an increased risk of incident diabetes, which is consistent with previous research [12–14, 27]. This implies that obesity and overweight are risk factors and that obesity in combination with MA contributes more to the development of T2DM. In our study, metabolically healthy participants with abdominal obesity had a higher incident risk of T2DM compared with the BMI-based MHO group. Obesity-related metabolic disorders have received increased attention as a result of their pervasiveness. Evidence suggests that abdominal obesity, particularly long-term abdominal obesity, is more closely associated with the development of T2DM [32, 33]. The adipose tissue secretes adipokines. Obese individuals have chronic inflammation as a result of increased proinflammatory adipokine levels and decreased anti-inflammatory adipokine levels [34]. Diabetes is a condition associated with inflammation, as it is characterized by chronic systemic inflammatory stimulation [35]. Therefore, it is important to identify MHO and MUO phenotypes as high-risk phenotypes for diabetes and implement early prevention measures. Periodic physical examination is critically important to monitor and manage physical health. It is recommended that the middle-aged and older adults should get regular physical examinations to detect any high-risk phenotypes, perform early diagnosis and timely interventions.

There were some limitations to our study. First, the sample size was small, and because the follow-up spans a long period, some older participants were lost to follow-up due to severe illness, hospitalisation, and other causes, and some blood samples were not collected; this caused problems with margins of missing follow-up data. The small number of MHO groups limited our statistical methods of choice. Second, although we were aware that diabetes had developed at some time point during the follow-up period, the precise time at which the change occurred was unknown. Thirdly, the participants in this study were mostly middle-aged and elderly rural residents from Northwest China. Inadequacies in the representativeness of the survey samples in comparison to the general population.

### Conclusion

At both the baseline and follow-up visits, more than half of the participants had an abnormal metabolic state. MUO was the most stable phenotype. The relative stability of the phenotypic groups was MUO > MUNW > MHNW > MHO. Diabetes risk differed between obese subgroups, with MHO and MUO phenotypes associated with an increased risk of developing T2DM, and metabolically healthy abdominal obesity participants having a higher incident risk of T2DM. In this view, there is an urgent need to encourage middle-aged and elderly people with MHO and MUO phenotypes to take weight-control measures to avoid constant weight gain, abnormal changes in metabolic state, and the occurrence of T2DM.
