# Supplementary material for: Obesity Metabolic Phenotype, Changes in Time and Risk of Diabetes Mellitus in an Observational Prospective Study on General Population
Source: Int J Public Health. 2022 Sep 29;67:1604986. doi: 10.3389/ijph.2022.1604986 (PMC9556707; doi:10.3389/ijph.2022.1604986)
Supplement: Supplementary file 1 [file DataSheet1.docx]

**Supplementary Table and Figure**

**Supplementary Table 1.** Percentage Change in Metabolic Obesity Phenotypes between Baseline and Follow-up. (Obesity Metabolic Phenotype, Changes in Time and Risk of Diabetes Mellitus in an Observational Prospective Study on General Population, China, and 2022).

|  | Baseline | Follow-up | P-value |
| --- | --- | --- | --- |
| Definitions based on BMI |  |  |  |
| MHNW | 316(22.68%) | 101(7.25%) | < 0.001 |
| MHO | 114(8.18%) | 90(6.46%) | 0.317 |
| MUNW | 500(35.89%) | 394(28.29%) | < 0.001 |
| MUO | 463(33.25%) | 808(58.00%) | < 0.001 |
| Definitions based on WC |  |  |  |
| MHNW | 338(24.26%) | 116(8.33%) | < 0.001 |
| MHO | 92(6.60%) | 75(5.38%) | 0.813 |
| MUNW | 600(43.08%) | 442(31.73%) | < 0.001 |
| MUO | 363(26.06%) | 760(54.56%) | < 0.001 |

P-value obtained in the chi-squared test. MHNW, metabolically healthy normal weight; MHO, metabolically healthy overweight/obese; MUNW, metabolically unhealthy normal weight; MUO, metabolically unhealthy overweight/obese; BMI, body mass index; WC, waist circumstance.

**
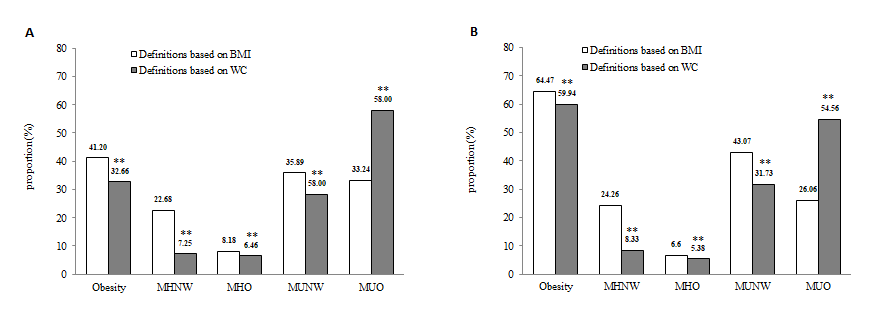
**

**Supplementary Figure 1.** Proportions of overweight/obese and different metabolic obesity phenotypes at baseline and follow-up. (Obesity Metabolic Phenotype, Changes in Time and Risk of Diabetes Mellitus in an Observational Prospective Study on General Population, China, and 2022). (A) Baseline. (B) Follow-up. In the graph, the column indicates the proportions. For the white columns, obesity was defined as a BMI of ≥ 24 kg/m^2^. For the grey columns, obesity was defined as WC≥ 90 cm for men and ≥ 85 cm for women. **P < 0.001 compared with the corresponding definition based on WC. MHNW, metabolically healthy normal weight; MHO, metabolically healthy overweight/obese; MUNW, metabolically unhealthy normal weight; MUO, metabolically unhealthy overweight/obese; BMI, body mass index; WC, waist circumstance.
